# Problem-solving therapy rather than treatment as usual for adults after self-harm: a pragmatic, feasibility, randomised controlled trial (the MIDSHIPS trial)

**DOI:** 10.1186/s40814-020-00668-0

**Published:** 2020-08-19

**Authors:** David Owens, Alexandra Wright-Hughes, Liz Graham, Paul Blenkiron, Kayleigh Burton, Michelle Collinson, Amanda Farrin, Simon Hatcher, Katie Martin, John O’Dwyer, Louise Pembroke, David Protheroe, Sandy Tubeuf, Allan House

**Affiliations:** 1 Leeds Institute of Health Sciences, University of Leeds, Worsley Building, Leeds, LS2 9NL UK; 2 Leeds Institute of Clinical Trials Research, Worsley Building, Leeds, LS2 9NL UK; 3 Academic Unit of Elderly Care and Rehabilitation, Bradford Institute for Health Research, Temple Bank House, Bradford, BD9 6RJ UK; 4 Tees, Esk & Wear Valleys NHS Foundation Trust, West Park Hospital, Edward Pease Way, Darlington, DL2 2TS UK; 5 Department of Psychiatry, University of Ottawa, 1145 Carling Avenue, Ottawa, Ontario K1Z 7 K4 Canada; 6 Leeds and York Partnership NHS Foundation Trust, 2150 Century Way, Thorpe Park, Leeds, LS15 8ZB UK; 7 Institute of Health and Society, Université Catholique de Louvain, Clos Chapelle-aux-Champs, 30, Box B1.30.01, B-1200 Brussels, Belgium

**Keywords:** Self-harm, Problem-solving therapy, Self-poisoning, Self-injury, Adults, Feasibility RCT

## Abstract

**Background:**

Non-fatal self-harm is one of the commonest reasons for adults’ emergency hospital attendance. Although strongly associated with fatal and non-fatal repetition, there is weak evidence about effective interventions—and no clear NICE guidance or clinical consensus concerning aftercare. We examined the practicability of a definitive trial to evaluate problem-solving therapy (PST) to reduce repetition of self-harm; MIDSHIPS is a single-centre, parallel-group, individually randomised controlled feasibility trial comparing treatment-as-usual (TAU) alone to TAU plus up to six sessions of brief problem-solving therapy (PST) with adults who had recently attended hospital because of self-harm. Objectives were to adapt the intervention for a UK setting, train therapists, recruit and randomise patients, deliver PST under supervision, and establish comparative outcomes, assessed blindly.

**Methods:**

We adapted the problem-solving intervention from an earlier trial and trained a mental-health nurse to deliver it. Adult patients attending the general hospital for self-harm were recruited while undergoing psychosocial assessment by the mental health team, and 62 were randomly allocated (32 TAU, 30 PST). The primary outcome assessed repeat hospital attendance due to further self-harm 6 months post-randomisation. Secondary outcomes included participant-reported outcomes and service use at 3 and 6 months post-randomisation.

**Results:**

The recruitment period had to be extended and 710 patients screened in order to establish a trial sample of the planned size (*N* = 62). A quarter of participants allocated to PST did not undertake the therapy offered; those who received PST attended a median of three sessions. Secondary outcomes were established for 49 (79%) participants at 6 months; all participants’ hospital records were retrieved. Repetition of self-harm leading to hospital presentation occurred in 19 of the 62 participants (30.6%, 95% CI 19.2%, 42.1%) within 6 months of randomisation. Promising differential rates of self-harm were observed with an event rate of 23.3% (95% CI 8.2%, 38.5%) in the PST arm; and 37.5% (95% CI 20.7%, 54.3%) in TAU. Economic findings were also encouraging, with a small QALY gain (0.0203) in the PST arm together with less reported use of the NHS in the PST arm (average £2120) than with TAU-only (£2878).

**Conclusions:**

The feasibility trial achieved its objectives despite considerable difficulties with recruitment—adapting the PST, training a therapist, recruiting patients who had recently self-harmed, delivering the therapy, and establishing primary and secondary outcomes. These data provide a robust platform for a definitive multicentre randomised controlled trial of brief problem-solving therapy after hospital attendance due to self-harm.

**Trial registration:**

Identification number and URL: ISRCTN54036115 http://www.isrctn.com/search?q=midships. Registered: 13 January 2012

## Background

Non-fatal self-harm is one of the commonest reasons for emergency hospital attendance in most countries and places a high demand on mental and physical health services; in the UK an estimated 150,000 people make over 220,000 hospital attendances each year [1]. Our definition of self-harm is the widely used one drawn from the Multicentre Study of Self-Harm in England and used in NICE guidance: intentional self-poisoning or self-injury, irrespective of degree of suicidal intent [2].

Working age adults represent the majority of hospital attendances due to self-harm [3, 4]. The excess of females is largely accounted for by young people in their teens and twenties [4]. Over 70% of episodes leading to hospital attendance are self-poisoning, with self-injury (most often cutting) accounting for most of the rest—although people who combine poisoning and injury in a single episode form a smaller (less than 8%) subgroup [5].

Over 15% of hospital-identified patients repeat within 1 year of an index episode [6, 7]. Repetition rates are similar among males and females; higher in middle adulthood than among younger or older patients [8]; and higher after self-injury than self-poisoning [5]. Most repetition is non-fatal although suicide, occurring in just under 1% of patients in the year following hospital discharge after self-harm, is around 50 times more likely than in the comparable general population [2, 7].

There is a poor evidence base about routine interventions following hospital attendance [9]. The latest Cochrane review found insufficient evidence to support case management or remote contact procedures but some benefit from psychotherapy comprising cognitive-behavioural therapy, problem-solving therapy or both—with Cochrane-GRADE evidence ratings only low-to-moderate [9]. NICE and UK professional bodies recognise the shortage of evidence for authoritative recommendations on treatment [10, 11]. As a consequence, assessment and aftercare following self-harm are highly inconsistent [12].

We therefore designed a large randomised controlled trial (RCT)—the *Multicentre Intervention Designed for Self-Harm using Interpersonal Problem Solving* (MIDSHIPS)—among patients recruited from general hospitals, using a brief cognitive-behavioural-based therapy—interpersonal problem-solving therapy (PST)—comparing outcome after PST plus treatment-as-usual (TAU) with outcome following TAU alone. The therapy was designed to be readily learned by mental health staff—such as nurses, occupational therapists, and social workers—and to be delivered in six sessions or fewer over 2 months. Although planning a multicentre RCT, we began by developing and undertaking a feasibility trial in one centre. The MIDSHIPS feasibility trial [13] aimed to: (1) establish the intervention; (2) train therapists; (3) recruit and randomise patients after hospital attendance due to a self-harm episode; (4) deliver the therapy under supervision; (5) monitor retention and adherence to PST and characterise TAU; (6) collect follow-up data; and (7) analyse the comparative outcomes between the two groups, to inform the sample size of a definitive trial.

## Methods

### Setting

The feasibility trial took place in one Mental Health Trust in Northern England that serves the cities of Leeds and York. Leeds has a population of over 700,000 and is one of UK’s largest city-regions; it has two large teaching hospitals each with its own Emergency Department (ED) service with overall management in a unified ED service. The nearby city of York, population 200,000 with its rural hinterland, has a single ED. In both cities, hospital policy held that all patients who attended due to self-harm should receive a psychosocial assessment by a clinician who had suitable training, time, and supervision for the task.

### The problem-solving therapy

Problem-solving therapy (PST) is a long-established brief and focused psychological intervention [14]. We (AH and LP) adapted our PST manual and training programme from ones used in self-harm trials in New Zealand [15, 16]. The MIDSHIPS PST has seven steps that are set out in detail in the PST training manual:

Step 1: Problem orientation

Step 2: Recognising and identifying problems

Step 3: Selecting and defining a clear problem

Step 4: Generating solutions

Step 5: Decision making

Step 6: Creating and implementing a SMART* action plan

Step 7: Reviewing progress

*Specific, measurable, achievable, relevant and time-bound

Liaison psychiatry researcher, AH, and service-user and activist in mental health, LP, also adapted the Client Workbook used in the New Zealand trials. This workbook takes the client through the problem-solving steps and includes: summarising sections; checklists of common experiences raised in PST; lists created in the sessions such as problems the client is currently experiencing, with potential solutions; diagrams and message boxes that illustrate and remind about the problem-solving approach; and homework—activities to practise between sessions. The workbook contains various sheets: for problem-lists, brainstorming, solution generation, and SMART (specific, measurable, achievable, relevant to the problem, and time-bound) action plans. For the present trial, the main additions were emphasis on planning for difficulty in implementing solutions, and on barriers to attendance at sessions and risks of dropping out of therapy.

### Recruiting therapists

We recruited two therapists—a mental health nurse and an occupational therapist. Their training in MIDSHIPS PST was delivered over two consecutive days at the University of Leeds by two liaison psychiatrists (SH and AH). Supervision was set up with a clinician-researcher (AH), to be delivered approximately fortnightly.

### Recruiting participants

Following meetings with senior clinicians in the ED and in the self-harm team, we decided against using ED clinicians to recruit participants. First, busy ED staff were not likely to be sufficiently focussed on either mental-health care or on recruitment to clinical trials. Second, the Mental Health Trust providing the clinical assessment of patients deemed it essential, for safety reasons, for any recruited patients to be risk-assessed before taking part. Direct recruitment by ED clinicians would have necessitated a separate, risk-assessing, clinical appointment for patients before the researcher would be allowed to arrange to meet them.

Consequently, we used only mental health clinicians in our two-stage recruitment procedure. At the routine psychosocial assessment resulting from an episode of self-harm, the mental health clinician (a mental health nurse or psychiatrist) completed a screening schedule (one double-sided sheet of paper) that established eligibility and then briefly introduced the trial to the eligible patient, passing on an A5-sized double-sided invitation card. Consent was sought at this stage only to an approach from a researcher who would later explain the trial more fully. This consent, together with contact details, was recorded on the invitation card—at the time of the clinical encounter or later: the invitation card could be folded and gummed shut to become a postcard with a prepaid address to the trial team. On receiving the card, from the clinicians or mailed by the patient, the MIDSHIPS researcher would contact the patient and arrange to meet, where full written consent to random allocation in the trial was sought following provision of the participant information sheet and a detailed discussion of what the research entailed. We also placed our MIDSHIPS invitation cards at a voluntary sector drop-in centre in Leeds to which people who had self-harmed were sometimes directed; in the event, none of the few expressions of interest were from people who had, recently enough to fit inclusion criteria, been to hospital as a consequence of self-harm.

### Inclusion and exclusion criteria

Inclusion criteria: aged 18 years or over, living in the Mental Health Trust’s catchment area, and attendance at the general hospital (at ED or as an in-patient) as a consequence of self-harm in the previous 6 weeks.

Exclusion criteria: receiving from another research project or clinical service an active intervention that would conflict with MIDSHIPS therapy, insufficient mental capacity to comply with trial requirements, insufficient proficiency in English to contribute to the data collection needed for the research, known risk of violence, researcher unable to make contact and randomise within 8 weeks of the most recent ‘index’ self-harm episode.

### Baseline data collection

The researcher arranged to meet potential participants at a variety of clinical locations but, due to safety considerations, not generally in their homes. The researcher collected, on case report forms designed for the MIDSHIPS trial, baseline information: basic personal information, details of the index self-harm event and consequent referral to hospital care, previous self-harm episodes, current physical and mental health, social and medical history, and current psychotropic medication. The researcher also administered two questionnaires: the Short Form 36 (SF-36) [17] health survey and a trial-specific healthcare resource-use questionnaire.

### Random allocation

Once consent and baseline data were collected, the researcher arranged random allocation using a 24-h randomisation system provided by Leeds Clinical Trials Research Unit. A computer-generated adaptive minimisation algorithm that incorporated a random element was used on a 1:1 basis to allocate participants either to PST plus TAU or to TAU alone. Minimisation was designed to balance the treatment groups for four characteristics: number of previous self-harm episodes leading to hospital treatment prior to baseline assessment (one, more than one); type of index episode (self-poisoning, self-injury, both combined); gender (male, female); and age (< 30, 30–59, 60 or over). Participants were told their treatment allocation as soon as possible after randomisation—by a researcher other than the one who conducted the assessment and took consent—in order to keep that researcher blind to allocation because of likely later involvement in follow-up data collection. To accomplish this blinded arrangement, the Trials Unit made two contacts: first, the problem-solving therapist was told of the allocation to the treatment arm, and details for making contact provided; second, a research assistant who worked not with the project team but with the hospital’s generic research support network (the nationally funded Comprehensive Local Research Network)—who was tasked with contacting each randomised patient, to pass on their allocation.

### Delivering the problem-solving therapy

Participants randomly allocated to the PST arm of the trial were invited to attend on six occasions for 1-h sessions with an additional ‘booster’ session, if wished-for, approximately 6 to 8 weeks after the main sessions. When appointments were not kept, the therapist made reasonable attempts to reschedule. Sessions were planned at 1- or 2-weekly intervals and were arranged at mental health service premises or at participants’ homes, according to patient preference and safety considerations. Participants were discharged from the PST only in the event of unscheduled failure to attend, or same-day cancellation of, three consecutive appointments.

### Ascertaining outcome and adherence data

#### Primary outcome

Repetition of self-harm leading to a hospital attendance was the planned primary outcome in the definitive RCT. Our participants gave permission for their attendance at local hospitals to be tracked and recorded by the research team at the end of the study. Researchers, blind to random allocation, collected these ED attendances and admissions by linkage of each participant to the electronic patient-record system for Leeds general hospitals, and by a manual search of the hospital’s record system for the York-recruited participants—confirming by scrutiny of hospital records in each case whether such attendances and admissions were due to self-harm. Consequently, every participant was accounted for with regard to the primary outcome.

#### Other outcome data

At 3 and 6 months post-randomisation, we collected by postal questionnaire, followed up by telephone if there was no response: self-reported episodes of self-harm, including ones not leading to hospital attendance; the SF-36 health survey; and a trial-specific schedule of healthcare resource used. TAU data were collected from the Trust's records by another researcher. In routine practice, many patients attending the hospitals in the trial because of self-harm are not offered aftercare, although some are followed up in general psychiatric outpatient clinics or referred to specialist services such as those dealing with drug and alcohol use; return of patients to the care of their general practitioner is the most usual form of TAU.

With Ethics Committee agreement, we issued participants with £10 shopping vouchers on up to three occasions: sent with the 3- and 6-month questionnaires, and on a final occasion after a telephone interview by an unblinded researcher—who asked about therapy appointments, other treatments received outside of the trial, and perceived burden and acceptability of the tasks concerned with follow-up data collection.

#### Adherence to problem-solving therapy

Adherence of therapists and participants to the PST was recorded in two ways: a record kept by therapists of sessions undertaken or missed, and by the researcher’s review of the therapy session notes for evidence of adherence to the manual—as judged by completion of workbook components such as problem lists, brainstorming sheets, solution generation sheets, and SMART action plans.

### Analysis

#### Sample size

As a feasibility trial, the current study was not powered to evaluate effectiveness [13]. We planned to recruit 60 participants over 6 months, randomised equally between TAU and PST + TAU, to allow for the investigation of parameters and outcomes for a definitive RCT.

#### Statistical analysis

As this was a feasibility study, formal hypothesis testing was not conducted, and descriptive analyses were performed on an intention-to-treat basis to provide estimates of key parameters for the definitive RCT. Summary statistics are reported for screening and recruitment, therapeutic delivery and adherence, characteristics of TAU, retention in treatment, and follow-up rates for outcome—collected from hospital records and self-reported. To inform the sample size calculation for the definitive trial, the occurrence and timing of repeat self-harm leading to hospital attendance are summarised according to trial arm at 6 months post-randomisation (with 95% confidence intervals).

### Health economics analysis

Data completeness of the SF-36 and the health service resource-use questionnaire were examined by the health economists (ST and JO’D), to inform the methods and cost perspective to be used for a definitive trial. Indicative costs for both arms were calculated through direct observation of the treatment provided as part of the trial, and through participant-reported resource use. Unit costs for resources were obtained from the British National Formulary, PSSRU unit costs of Health and Social Care 2013 [18], and the Department of Health’s National Schedule of Reference Costs [19]. Utilities were generated from the SF-6D preference-based measure based on the participants’ responses to the SF-36 questionnaire at each time point [20]. Utilities were then converted to QALYs, multiplying utilities by the time spent in each state, with quality of life linearly interpolated for the periods between the three observations provided in the feasibility study (baseline, 3 and 6 months).

### Ethical and governance considerations

Ethical approval for the feasibility trial was granted by the Leeds West Research Ethics Committee (Reference 12/YH/022); research governance approval was from the UK National Institute for Health Research’s (NIHR) Coordinated System for gaining NHS Permissions (CSP 88012); and health service permission to undertake the research was granted by the local mental-health and acute teaching-hospital Trusts’ research and development departments. We set up a Trial Management Group that met regularly to run the trial and an independent Trial Steering Committee to provide independent and scientific oversight of the research [13].

## Results

During an extended 13-month recruitment period, from October 2012 to October 2013, 710 patients were screened, of whom 392 (55%) were deemed eligible, and 62 (16% of eligible) were recruited. Almost half (48%) of the ineligibility was because clinical staff thought the patient was a risk of violence—to therapy or research staff. Other common reasons for clinical staff to exclude patients at this early stage were: lack of certainty about whether there had been recent self-harm as defined (23%), with alcohol and drug misuse sometimes raising uncertainties; concurrent involvement in other research or with a clinical service that would exclude their recruitment (14%); and not living in the catchment area (10%). Of the patients screened as eligible, 24% (94/392) did not have the trial introduced to them. Although some of these patients (34/94) were deemed too distressed or unwell, or were about to move from the catchment area, or had other understandable reasons for not being approached, it was not clear why the other 60 were not approached by clinicians; the research team scrutinised these patients’ records soon after these attendances and sent letters inviting response to 55 of these 60 people; no-one replied.

### Recruitment and baseline

**Fig. 1:**
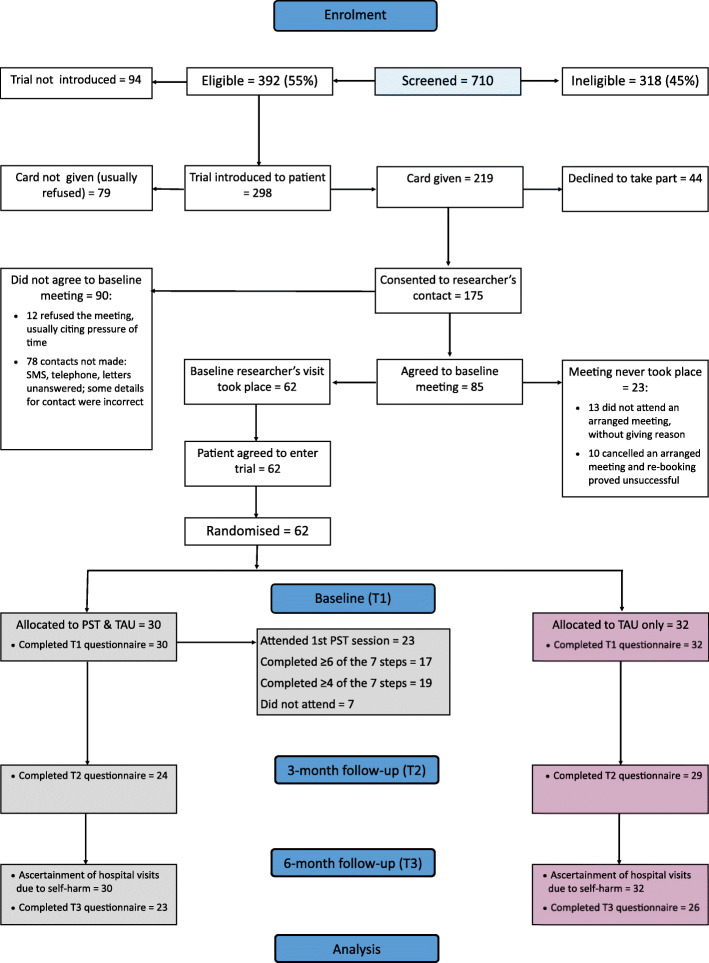
Flow of potential and actual participants through the study

**Fig. 2:**
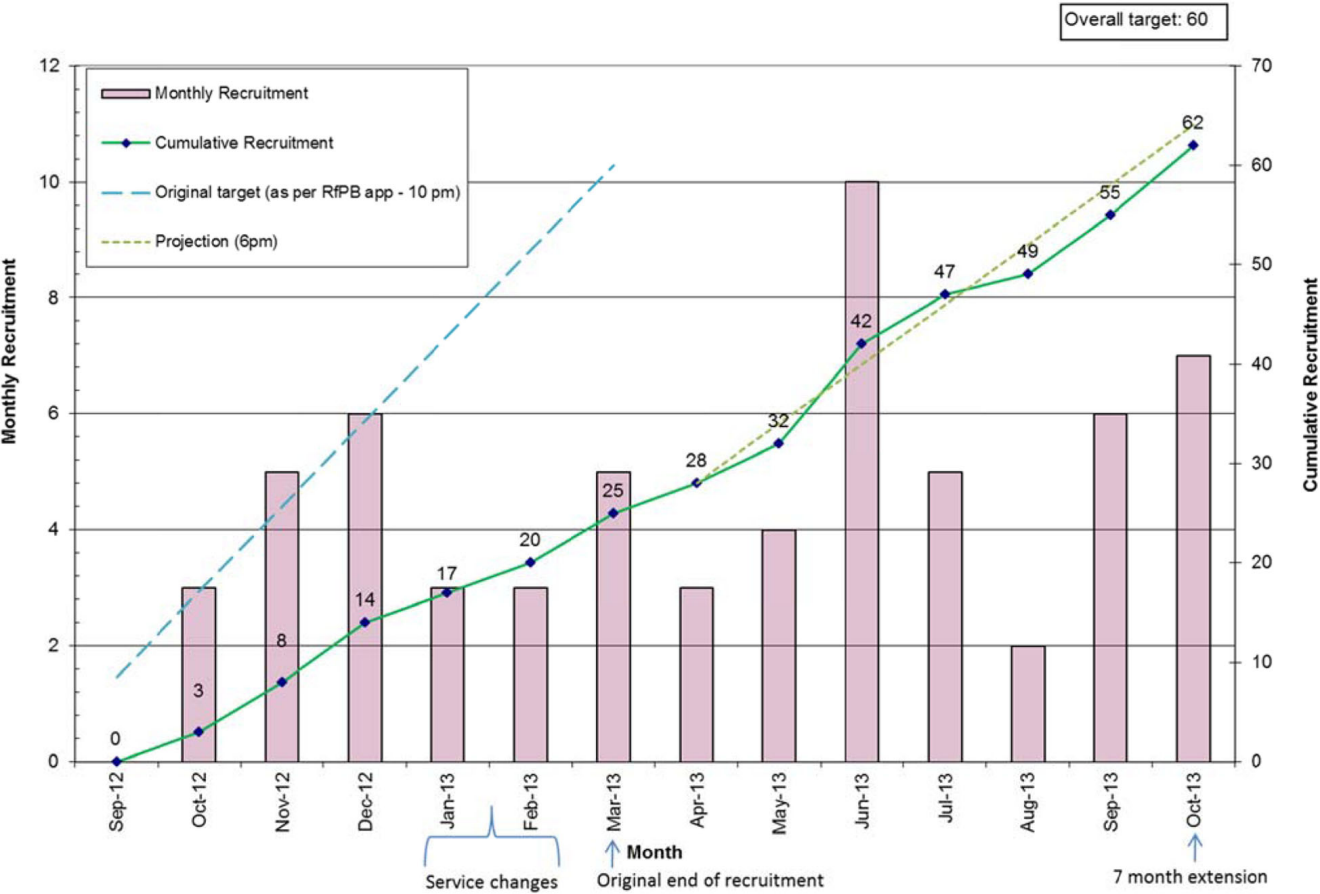
Monthly and cumulative recruitment to the feasibility trial

Recruitment to the trial is set out in Fig. 1. The rate of screening by clinicians was markedly lower than we had predicted (around 60 rather than 190 per month) and the proportion of those eligible who were recruited (62/392) was also below our forecast (16% rather than 20%). We had correctly anticipated many barriers to recruitment, and we made repeated attempts to set up successful baseline interviews. In all, research staff made 1359 attempts at contacting the 175 patients consenting to researcher contact regarding their inclusion in the trial. With recruitment to the trial well below its proposed trajectory, we extended the trial’s recruitment phase from 6 to 13 months (Fig. 2), and we extended our initial recruitment catchment of Leeds to include the neighbouring city of York—which was part of the same mental health service Trust.

The trial recruited 62 participants who were randomly allocated, 32 to TAU and 30 to TAU plus PST (Fig. 1). Eight of the 62 participants were recruited from York, allocated equally to trial arms. Just 6 baseline interviews were at patients’ homes, with the rest at health-service premises, mainly hospitals. Age and gender profiles were reasonably similar among screened patients, eligible patients, those approached, and those enrolled. Trial participants were a little older, were more often female, and included more patients who had self-injured rather than self-poisoned: screened mean (median) age was 34.1 (32.0) years compared with 35.2 (34.7) in the trial; the proportion of females was 58% among those screened and 65% in the trial; and in those screened, the proportion who self-poisoned, self-injured, or combined both methods were respectively 77%, 15%, and 8%, with the corresponding proportions in the trial 65%, 19%, and 16%.

### Characteristics of the sample

Baseline socio-demographic and clinical characteristics (Table 1) were similar across the two trial arms. The trial sample had a higher than expected proportion who had combined self-injury with self-poisoning but otherwise the proportions of poisoning and injury were broadly similar to hospital observational data. There were high incidences of previous self-harm (79%) and of treatment for mental health problems (84%). Alcohol and substance use were typical of self-harm patient populations.

**Table 1:** Socio-demographic and clinical characteristics of the patients in the two treatment arms

	Problem-solving and treatment as usual (*N* = 30)	Treatment as usual (*N* = 32)	Total (*N* = 62)
Female	19 (63%)	21 (66%)	40 (65%)
Age			
Mean (SD)	34.8 (13.9)	35.6 (14.0)	35.2 (13.8)
Median (range)	35 (18 to 62)	35.5 (19 to 65)	35 (18 to 65)
Age group			
<30 years	13 (43%)	15 (47%)	28 (45%)
30 to 59 years	15 (50%)	16 (50%)	31 (50%)
≥ 60 years	2 (7%)	1 (3%)	3 (5%)
Type of index event			
Self-poisoning	20 (67%)	20 (62%)	40 (65%)
Self-injury	6 (20%)	6 (19%)	12 (19%)
Combined	4 (13%)	6 (19%)	10 (16%)
Self-harm before index event			
No previous episodes	7 (23%)	7 (22%)	14 (23%)
One or more episode	23 (77%)	25 (78%)	48 (77%)
Previous treatment for mental health problem	25 (83%)	27 (84%)	52 (84%)
Living alone	10 (33%)	9 (28%)	19 (31%)
Alcohol consumption			
Never	2 (7%)	3 (9%)	5 (8%)
Once a week or less	17 (56%)	14 (44%)	31 (50%)
Twice a week or more	11 (37%)	15 (47%)	26 (42%)
Substance use			
Never	13 (43%)	12 (38%)	25 (40%)

Values are numbers (%) unless specified

### Trial delivery and adherence

In no case did the research team in advance deem there to be a safety concern over the conduct of an interview. In two cases, the researcher had concerns, at the interview, about the participant’s own safety and followed the established risk protocol, without needing to trigger a contact with health service agencies. No incidents arose following baseline interviews.

### Therapeutic delivery

Two therapists were successfully trained in the PST intervention but one left early in the treatment phase after delivering therapy, under supervision, to one participant. All PST (excluding 3 sessions for that one participant) was delivered by the remaining therapist. Supervision was regular, with the therapist receiving 25 face-to-face supervision sessions, approximately two-weekly, each lasting on average 54 min. During supervision, each new participant was discussed in detail, as were the other participants currently in therapy.

#### Problem-solving therapy

Of the 30 participants allocated to PST, 13/30 (43%) attended all planned sessions, another 10 (33%) attended at least one session, and 7 (23%) attended no sessions. The 23 (77%) participants attending at least one session attended a median of three, with median time from first to last session of 59 days (range 0 to 154), median time from randomisation to last session 84 days (26 to 197), and median duration of treatment 5.2 h (0.8 to 7.9). All attendees were supplied with a workbook, 14/23 (61%) completed all 7 therapy steps, 17 (74%) completed 6 steps or more, and 19 (83%) completed 4 or more of the steps. The independent review of adherence reported evidence that all 23 participants had been given a workbook; 22 (96%) had a problem list; 20 (87%) had defined a problem; and 17 (74%) had a brainstorm of possible solutions and a SMART action plan. Overall, 16 (70%) participants had evidence of compliance to all five of these items, 18 (78%) to at least four, and 20 (87%) to at least three.

#### Receipt of treatment as usual

Data were collected from mental-health service records for all 62 participants; some form of TAU was received by 38 (61%) of participants: 16 (53%) of PST-arm participants and 22 (69%) of TAU-arm participants. There were 797 TAU contacts (Table 2): 66% face to face and 34% over the telephone, 94% of them individual rather than group sessions. The contacts varied widely—from short telephone calls made to remind patients about impending appointments, to follow-up psychosocial assessments resulting from the self-harm episode, and to participation in forms of therapy such as group-art; a large majority of the contacts were for administrative and follow-up assessment rather than therapeutic reasons (Table 2).

**Table 2:** Treatment as usual accessed and received during follow-up

	Problem-solving and treatment as usual (*N* = 30)	Treatment as usual (*N* = 32)
Contacts and visits to facilities		
Community Mental Health Team contacts	235	470
Emergency Department	8	13
Psychiatry clinic	10	19
Clinical Psychology clinic	0	27
Substance use unit	5	3
Other	1	6
**Total**	**259**	**538**
Nature of contacts or visits		
Administration and follow-up, not therapy	226	527
Cognitive behavioural therapy	1	0
Dialectical behavioural therapy	1	1
Counselling	1	0
Group therapy	21	6
Other	9	4
**Total**	**259**	**538**

Values are numbers of episodes

### Data collection

Every participant provided all required baseline data. The trial had a high response rate for return of questionnaire booklets: 86% (53/62) at 3 months and 79% (49/62) at 6 months, with similar follow-up rates observed across trial arms (Fig. 1). Most of the responses (52/53 and 42/49) were returned by post; the greater use of telephone calls at 6 months was largely due to pressure on researchers to adhere to time-limits as the project approached its end. Questions on the forms were answered adequately: only 9 of the 94 forms had missing items—usually only one with a maximum of three items missing. Hospital attendance data were available for all participants.

### Statistical outcomes

**Fig. 3:**
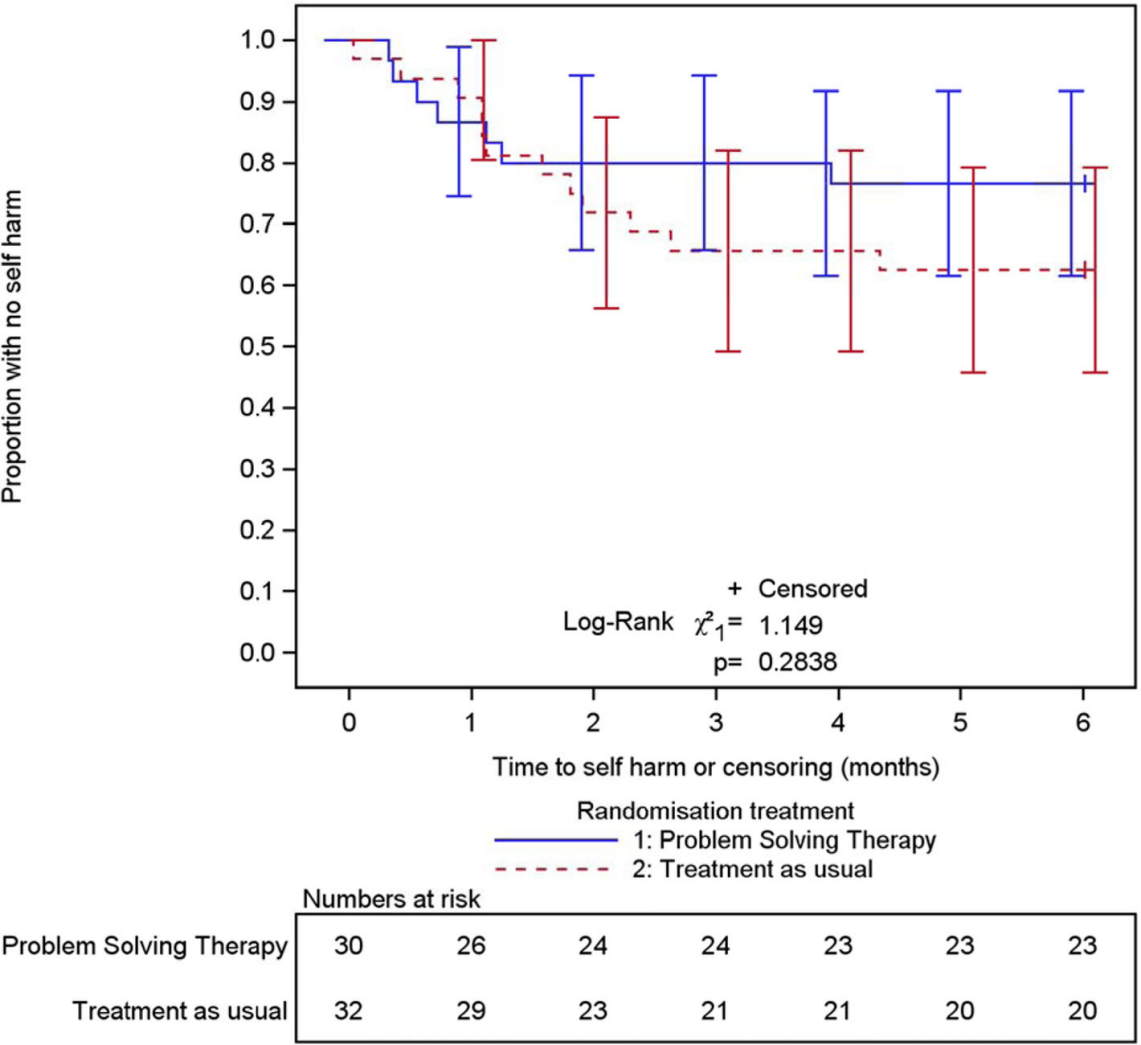
Kaplan-Meier plot of time to self-harm event; vertical bars are 95% confidence intervals

Further hospital attendances due to repeat self-harm were our candidate for primary outcome in a multicentre trial: the findings here are set out in Table 3. Repetition of self-harm leading to hospital presentation within 6 months of randomisation occurred in 19 of the 62 participants (30.6%, 95% CI 19.2%, 42.1%). We observed promising differential rates of self-harm: 23.3% (7/30, 95% CI 8.2 to 38.5%) of participants repeated 18 times in the PST arm; and 37.5% (12/32, 95% CI 20.7 to 54.3%) repeated 44 times in the TAU-only arm. The time to repetition, however, showed a more complex pattern, with speedier initial repetition among participants randomised to problem-solving therapy (Table 3, Fig. 3). The overall proportion repeating among those who had no previous history of self-harm was considerably lower (2/13, 15%) than in those with a history of earlier self-harm (17/49, 35%).

**Table 3:** Return to hospital due to repetition of self-harm within 6 months, according to trial arm

	Problem-solving and treatment as usual (*N* = 30)	Treatment as usual (*N* = 32)	Total (*N* = 62)
Repeated and attended hospital	7 (23%)	12 (38%)	19 (31%)
In participants with an event:	*n* = 7	*n* = 12	*n* = 19
Mean time in months (SD) to first repeat event	1.2 (1.27)	1.6 (1.14)	1.4 (1.17)
Median time in months (IQR) to first repeat event	0.7 (0.4 to 1.2)	1.3 (1.0 to 2.1)	1.1 (0.6 to 1.9)
Mean number (SD) of events per participant	2.6 (1.40)	3.7 (2.74)	3.3 (2.35)
Number of repeat events	18	44	62
Type of repeat event			
Self-poisoning	12 (67%)	36 (82%)	48 (77%)
Self-injury	3 (17%)	3 (7%)	6 (10%)
Combined	1 (6%)	2 (5%)	3 (6%)
Detail missing	2 (11%)	3 (7%)	5 (8%)
Mean number (SD) of events per participant (all 62 participants)	0.6 (1.28)	1.4 (2.43)	1.0 (1.98)

Values are numbers (%) unless specified

Self-report of further self-harm was available for 54 (87%) participants who responded to at least one follow-up questionnaire. Further self-harm was reported in the 6 months following randomisation for 32% (8/25) of participants allocated to PST and 59% (17/29) of participants allocated to TAU. However 4/25 responses in the PST arm were incomplete, covering either months 1–3 or 4–6 post-randomisation.

As almost all PST sessions were delivered by one therapist, it was not possible to investigate the therapist clustering effect.

### Safety

No related unexpected serious adverse events (RU-SAEs) were reported. One death (a TAU participant) was reported after the 6-month follow-up period. A reassuring trend of fewer SAEs, defined as hospital attendances, was observed in the PST group with 26 attendances in 12 (40%) PST participants and 89 attendances in 20 (63%) TAU-only participants. Differential rates of hospital attendance were corroborated by self-reported health-resource use data at 3 months, with PST-arm participants reporting fewer ED visits than TAU-only participants (13% vs 58%). Similar self-reported hospital attendance rates were reported at 6 months for the preceding 3-month period (30% vs 27%).

### Health economics

Complete data for the health economics analysis were available for a sample of 48 (77%) participants (22 in the problem-solving arm, 26 in the treatment-as-usual arm); there were also some participants who responded at one of the two time periods. We generated the SF-6D preference-based measure from the SF-36 and found a higher (better) score for participants in the PST + TAU arm at all time points, including baseline (see Table 4) although differences between the two arms were never significant. When transforming the SF-6D to QALYs, we found a gain of 0.0203 between the arms over the 6 month follow-up—equivalent to one additional week of perfect health for those receiving problem-solving therapy over a year.

**Table 4:** SF-6D according to treatment arm; values are SF-6D scores—the preference-based measure derived from the SF-36 health survey

Treatment arm	Timepoint	Observations	Mean	Std Dev.	Min. per patient	Max. per patient
PST + TAU	Baseline	22	0.5877	0.1066	0.3830	0.8280
	3 months	22	0.6237	0.0907	0.4310	0.8410
	6 months	22	0.6411	0.1104	0.3700	0.8520
TAU	Baseline	26	0.5520	0.8695	0.3160	0.6790
	3 months	26	0.5731	0.0952	0.4130	0.8100
	6 months	26	0.5779	0.1164	0.3890	0.8870

The average total reported service use (NHS) was estimated at £1501 (SD 1440) at 3 months and £2120 (SD 2116) at 6 months in the problem-solving arm compared with £3238 (SD 3731) at 3 months and £2878 (SD 3721) at 6 months for those receiving only TAU. Both arms received TAU with costs estimated at £1790 (SD 1934) in the PST arm versus £2800 (SD 4094) in the TAU arm. We estimated the cost of delivering PST assuming that participants received six 1-h weekly sessions and were offered an optional ‘booster’ session. We also estimated the actual cost of receiving PST, including the trial data on session attendance and duration of the session, booster session attendance, and any other contacts by the participant with the therapist (phone calls, duration of phone calls, face-to-face meetings, and text messages). The expected cost of the therapy was £343 if the participant took up the optional booster session (versus £184 in the observed trial data) and £294 (versus £171) without a booster session.

## Discussion

Objectives of the feasibility trial were met. The problem-solving intervention was adapted from its earlier New Zealand version, and we deemed it a practicable intervention for hospital patients who had self-harmed in England. We successfully recruited and trained two part-time therapists from mental health staff in Leeds—although our feasibility trial, in the event, depended on just one of those therapists.

### Recruitment

Recruitment to the trial proved a challenge and fell behind schedule. Our planning estimates for identification, introduction, and recruitment were mixed in their accuracy. We only modestly overestimated that 20% of those who were eligible would give their consent; in fact it was 16%. On the other hand, only around one-third of the expected number of patients (around 60 rather than 190 each month) was screened.

The main reason for failure to screen was a local organisational one. Although good personal relations with the clinicians were maintained, our research was the victim of unfortunate timing. On the week when screening and recruitment began, an unanticipated restructure of the clinical teams was announced—with the self-harm team to be amalgamated with a generic acute-care ‘Crisis’ team. The actual changes in practice were enacted after a 3-month period: the consequences were that, throughout the trial, the screening and recruitment was undertaken by an unsettled and clinically disparate team of health-service professionals, many of whom were not experienced in self-harm work. We were thereby unable to count on as much commitment to the trial and its procedures as we had expected from the developmental work undertaken with the much-smaller, designated self-harm team.

For 48% of the patients screened and found to be ineligible for the trial, the reason was categorised as ‘known risk of violence’. In the health service, a modest proportion of patients would be ruled out of individual therapy because of a clear propensity for violence, but clinicians in the study were using a low threshold for such concerns. We sought repeatedly to emphasise to clinical staff that we were intending to mimic the non-trial provision of PST should it become available as a routine service, and people who were known to have taken part in a past and non-severe violent episode would not necessarily be excluded from the therapy and therefore ought not to be excluded from the trial.

We know that 60 patients were formally screened by clinical staff and deemed eligible to take part but did not have the trial introduced to them for unrecorded reasons. NHS-employed staff examined the ED records of these patients soon after they were discharged from the hospital and sent letters introducing the trial to 55 of them. Not one response was received and we concluded that there was little chance of recruitment if the research was not broached as part of the clinical care of the current episode. Similarly, many patients took away the addressed, pre-paid study card but few were returned; nearly all our initial recruitment was confirmed by patients at the point of contact with the clinician who had introduced the idea of taking part in the trial.

We had expected that some patients, although they had agreed to learn more about the trial through meeting with the researcher, would fail to respond to our email or telephone invitations. What we had not expected was so often to find that the contact details supplied by the patients contained errors. Having written consent to researcher-contact meant that we were sometimes able to correct these errors from hospital records but there remained many occasions where we were unable to make the contact. We would, in future, pay more attention to the method of collection of contact details—possibly to include details, where appropriate and acceptable, of a relative or other significant person—should a contact problem arise.

In more than a quarter of the cases where the researcher and patient arranged to meet to discuss participation in the trial (23/85), the meeting never took place. On more than half of these occasions, and without explanation, the patient failed to attend, or to be at home, or to open the door—and a further appointment did not prove possible.

### Trial participants and the two study arms

In clinical practice, greater uptake of talking therapies by women than by men is an established finding [21] so we had expected the modest excess of females in the trial compared with the proportion of females among the patients screened. We recruited a higher-than-expected proportion of patients who had self-harmed by combining self-injury with self-poisoning. If people who have used combined methods of self-harm are preferentially recruited, there are reasons to regard it as a good thing: these patients have less favourable outcomes than those who have only self-poisoned or only self-injured [5].

### Data collection

We achieved complete recording of baseline data from trial participants. When a trial’s participants are people who have self-harmed, where levels of clinical engagement and cooperation are uncertain, fears of poor ascertainment of follow-up events are a realistic concern for a research project. Here, researchers blind to the treatment allocation were able to identify all ED attendances for further episodes of self-harm over the follow-up 6 months by interrogating the record systems of the EDs in Leeds and York. We explored the possibility of using instead NHS Digital Hospital Episode Statistics for the identification of these attendances, but the processes were not sufficiently in place for us to gain permission and access these data; we know from our recent clinical trial of self-harm intervention in young people following self-harm [22, 23] that it will be possible to use such a procedure effectively in a future definitive trial similar to MIDSHIPS.

Non-return of follow-up questionnaires led to a reminder telephone contact from a researcher, who would collect questionnaire data over the telephone should postal return seem unlikely. The follow-up information sought in writing from participants was kept strictly to the minimum data deemed necessary for ascertainment of the trial’s important outcomes, in order to minimise the participants’ time and effort. As a result, rates of completion and return of secondary outcome data were high: 85% at 3 months and 79% at 6 months—higher than in many trials and perhaps unexpectedly so among a population of hospital patients who had self-harmed.

### Delivery and adherence

Three-quarters (74%) of the participants who engaged in the therapy completed six or seven of the seven problem-solving steps—confirmed by the therapist’s account and corroborated by completed worksheets and task-lists. Some of those who finished therapy before this stage did so in negotiation with the therapist: the participant considered that he or she had made gains from PST therapy and did not wish further attendance. That leaves, however, around one in five participants who began but did not persist with the therapy to a clinically meaningful extent.

### Outcomes

We found repetition rates for self-harm that were in the direction of benefit for PST. Although we found substantial effect sizes, for proportions repeating and for timing of repetition, precision was low as the study was not powered to detect a difference between arms. The follow-up data containing self-reports of further self-harm events also suggested some promise from the PST.

### Service-use and economic analysis

Our economic analysis suggested that PST was less costly than TAU alone, with the difference mainly coming from less self-reported service use and lower use of TAU in patients allocated to PST. Intervention with PST did point towards some benefits in quality-of-life measures but these findings were modest in effect size and not statistically significant. We observed an inherent problem in economic analysis when TAU is used in both arms: it can be unclear whether self-reported resource use is clearly distinct from data collected for TAU as part of the trial, and there is a risk of double counting some health care use.

### CONSORT compliance

This written account of the MIDSHIPS trial complies with the CONSORT statement’s extension to randomised pilot and feasibility trials [24].

## Conclusions

We found that it was feasible to produce a problem-solving therapeutic intervention well suited to a wide range of patients who had attended hospital after self-harm. The therapy was readily and quickly taught, and delivered without significant problems to people who were willing to accept an offer of therapy. Recruitment to the trial was slower than expected but we learned a good deal about how to avoid or minimise, in a future trial, a number of the impediments to the recruiting of those who had recently self-harmed—and we substantially improved recruitment in the second half of the study period. In line with the difficulties described here, we recruited a lower proportion of the eligible patients than did comparable trials in self-harm. A group therapy trial in Ireland of problem-solving skills training randomised 38% of the eligible patients [25]. In New Zealand, every eligible patient, in a trial using a care package that included problem-solving therapy, was randomised—because the trial used a Zelen design in which randomisation preceded consent; 46% of those randomised then gave their consent to their inclusion in the trial [16].

We successfully determined the primary outcome—repeat self-harm followed by hospital attendance—for all participants without expending excessive researchers’ time. We understand that we would be able in future to make such ascertainment more effective by using routinely collected data at a national level, using NHS Digital; such a step would be less labour-intensive and would have the advantage of identifying repeat episodes at hospitals other than the ones in the recruitment centres. Secondary outcome data and basic health economic measures showed some shortfall in their ascertainment but, by keeping the time and effort required for participants to a minimum, we achieved completion rates that stand up well against trials of complex interventions in general. The comparison of repetition rates across the two arms of the study, in favour of the problem-solving intervention, provides an encouraging preliminary finding.

Taken together, we believe that the experience and findings of the MIDSHIPS feasibility trial provide a robust platform for a definitive multicentre randomised controlled trial of brief problem-solving therapy, in the recruitment setting of the psychosocial assessment that should routinely result from hospital attendance due to self-harm.

## Data Availability

Individual participant data (with any relevant supporting material, e.g. data dictionary, protocol, statistical analysis plan) for all trial participants (excluding any trial-specific participant opt-outs) will be made available for secondary research purposes according to a controlled access approach. Data will only be made available in such a way that data recipients cannot identify individuals by any reasonably likely means, and we will only share data for projects that are clearly in the public interest and compatible with the original purpose of the data processing. No data will be released before an appropriate agreement is in place setting out the conditions of release. Requests to access trial data should be made to CTRU-DataAccess@leeds.ac.uk in the first instance.

## Abbreviations

ED: Emergency Department
NICE: National Institute for Health and Care Excellence
NIHR: National Institute for Health Research
PST: Problem solving therapy
QALY: Quality adjusted life year
RCT: Randomised controlled trial
TAU: Treatment as usual
